# A Computational Approach to Evaluating Empirical Antibiotic Coverage for Gram-Negative Bloodstream Infections in Pediatric Febrile Neutropenia

**DOI:** 10.3390/antibiotics15020192

**Published:** 2026-02-10

**Authors:** Francesca Cappozzo, Marcello Mariani, Emanuela Caci, Roberto Bandettini, Alessio Mesini, Erica Ricci, Carolina Saffioti, Carlo Dufour, Maura Faraci, Alberto Garaventa, Claudia Milanaccio, Francesca Bagnasco, Martina Toto, Elio Castagnola

**Affiliations:** 1 Department of Neuroscience, Rehabilitation, Ophthalmology, Genetics, Maternal and Child Health (DINOGMI), University of Genoa, 16132 Genoa, Italy; 2 Infectious Diseases Unit, IRCCS Istituto Giannina Gaslini, 16147 Genoa, Italy; 3 Laboratory of Microbiology, IRCCS Istituto Giannina Gaslini, 16147 Genoa, Italy; 4 Hematology-Oncology and HSCT Unit, IRCCS Istituto Giannina Gaslini, 16147 Genoa, Italy; 5 Epidemiology and Biostatistics Unit, IRCCS Istituto Giannina Gaslini, 16147 Genoa, Italy; 6 Department of Surgical Sciences, University of Cagliari, 09123 Cagliari, Italy

**Keywords:** febrile neutropenia, antibiotic therapy, computational script

## Abstract

**Background**: Empirical antibacterial therapy for febrile neutropenia requires adaptation to local epidemiology, a process that is often complex, time-consuming, and prone to human error. This study aims to address this challenge by developing a practical, data-driven tool to efficiently evaluate and adapt treatment protocols. **Methods**: We developed a novel, open-source computational script in Python (version 3.10), aided by large language models for code revision, to analyze antibiotic susceptibility data. The script was validated using a retrospective dataset of 237 Gram-negative bloodstream infections (BSIs) from 2015 to 2024 in cancer or hematopoietic stem cell transplant recipients at a tertiary care pediatric hospital in Italy. The script calculates efficacy metrics for both single agents and two-drug combinations. **Results**: Among the Gram-negative BSI strains analyzed, meropenem monotherapy demonstrated the highest efficacy (median 95.4%). In contrast, piperacillin/tazobactam and cefepime showed lower efficacy (80.3% and 81.8%, respectively). On the contrary, combination therapy, particularly with amikacin, significantly increased the efficacy of beta-lactams, elevating their effectiveness to a level comparable to meropenem. **Conclusions**: The developed script is a valuable tool for antimicrobial stewardship programs, offering a rapid and accessible method to validate international guidelines against local epidemiological data. While meropenem shows high efficacy, its broad use should be limited to prevent resistance. The combination of piperacillin–tazobactam and amikacin is identified as a robust and effective empirical treatment choice.

## 1. Introduction

Empirical antibacterial therapy is now a cornerstone of the treatment of fever in neutropenic patients receiving antineoplastic chemotherapy or a conditioning regimen for hematopoietic stem cell transplantation (HSCT). This approach, widely adopted since the latter part of the 20th century, has been proven to significantly reduce mortality from bloodstream infections (BSIs), particularly those caused by Gram-negative rods [1,2]. In the pediatric population, fever occurs in about one-third of neutropenic periods, with differences according to underlying conditions, but bacteremia is diagnosed in less than 20% of episodes, depending on the underlying disease and treatment phase [3,4,5]. Recent data show that Gram-negatives now account for approximately 50% of these BSI episodes [6,7,8]. Moreover, in recent years there has also been a significant increase in antibiotic-resistant Gram-negative infections, posing a substantial challenge in the management of children experiencing febrile neutropenia [6,9]. International guidelines [10,11] for the initial empirical treatment of febrile neutropenia in children recommend monotherapy with an antipseudomonal beta-lactam (e.g., piperacillin–tazobactam or cefepime) or a carbapenem (predominantly meropenem). According to guidelines, combination therapy with an aminoglycoside should be reserved for clinically unstable patients, those with suspected resistant infections, or in centers with high rates of resistant pathogens. A key recommendation is to tailor antimicrobial selection based on local epidemiological data, but developing locally tailored empirical therapy protocols can be a complex and time-consuming process, as it requires analyzing large datasets. This process is not only time-consuming and resource-intensive but also inherently susceptible to human error [12,13].

The reliance on manual spreadsheet management for antimicrobial stewardship presents, in fact, several critical vulnerabilities rooted in both statistical error rates and human cognitive limitations. Systematic reviews highlighted that manual Medical Record Abstraction (MRA) is associated with a pooled error rate of approximately 6.57%, a magnitude sufficient to compromise the statistical power of clinical trials [14]. This vulnerability is exacerbated by the specific nature of the data entry task; indeed, the common practice of “visual checking”—where a single operator verifies data against the source—has been shown to be statistically ineffective, resulting in 2958% more errors than double-entry systems [15]. Furthermore, the complexity of microbiological data adds another layer of risk. While overall clinical database error rates may be relatively low (~2.8%), text-based fields have been observed to be significantly more prone to transcription errors compared to numerical fields [16]. Since antibiograms rely heavily on the correct textual classification of species (e.g., *Klebsiella pneumoniae* vs. *Klebsiella oxytoca*), manual processing is inherently fragile. Finally, the sheer volume of data in retrospective cohorts introduces the physiological barrier of the “vigilance decrement”. Sustained attention tasks requiring multiple visual fixations inevitably lead to cognitive fatigue and processing delays [17]. In a clinical setting, reviewing hundreds of susceptibility rows induces this fatigue, making error inevitable regardless of the clinician’s expertise. Consequently, the transition to a computational workflow is not merely a matter of speed, but a necessary step to mitigate the biological and procedural limitations of manual curation.

The increasing complexity of antimicrobial resistance patterns underscores the need for more time-efficient and precise analytical approaches. To bridge the gap between international guidelines and the specific needs of a tertiary care pediatric hospital, we developed a novel computational script in Python [18] for the analysis of antimicrobial efficacy, to provide a practical data-driven method for evaluating and adapting guideline-derived treatment protocols. The script was tested on a BSI dataset from the IRCCS Istituto Giannina Gaslini, Genoa, Italy, to evaluate the adequacy of antibiotics for their empirical use in neutropenic cancer or HSCT patients, in the presence of Gram-negative BSIs.

## 2. Results

A total of 459 strains were obtained in patients with BSIs during the study period. Gram-positives accounted for 222 (48.4%), while Gram-negatives represented the most frequently isolated pathogens during the entire study period, accounting for 237 strains (51.6%). The family of *Enterobacterales* accounted for about 63% of total isolates, and *Escherichia coli* had the highest prevalence, while *Pseudomonas aeruginosa* accounted for 8.7% (n = 40) of strains. Table 1 summarizes the data on Gram-negatives that were subsequently used for the study.

Table 2 summarizes the efficacy results for selected antibiotics evaluated as monotherapies or in combinations. Among monotherapies, meropenem demonstrated the highest efficacy, with a median of 95.4% of tested strains resulting in susceptibility. Other agents indicated in guidelines showed lower efficacy values: cefepime 81.8%, and piperacillin/tazobactam 80.3%. For the other drugs, the proportions of efficacy were even lower, except for amikacin, which showed a median efficacy of 93.5%. The analysis of combination therapies’ efficacy showed a negligible increase for meropenem + amikacin in comparison to monotherapy, while there was a significant increase in efficacy for all other beta-lactams, especially when combined with amikacin, ranging from 93.5% to 95.4%, compared to their respective single agents (*p* < 0.001).

Mortality was 5% (12/237), and resistance to carbapenems was observed in 25% (3/12) of patients who died.

### Computational Validation and Efficiency

The validation analysis confirmed 100% concordance between the Python script and the standard R-based analysis (Table 3). No discrepancies were observed in the efficacy point estimates or the confidence interval boundaries across all tested antibiotic combinations (discrepancy rate = 0.00%).

However, a substantial difference was observed in workflow efficiency. The Standard Workflow required significant manual pre-processing within the spreadsheet to filter skin contaminants and assign Gram-stain classifications before importing data into R. Furthermore, generating Jeffreys intervals in R required specific coding expertise. In contrast, the Python script automated these pre-analytical steps via its internal dictionary-mapping algorithm. Consequently, the total processing time was reduced from approximately 150 min (Standard Workflow) to about 30 s (Python script), representing a >99% reduction in workload while maintaining identical statistical rigor.

## 3. Discussion

In this study, we applied a self-developed, open-source script to efficiently process the results of antimicrobial susceptibility tests from 237 Gram-negative strains isolated in BSIs in children receiving antineoplastic chemotherapy or HSCT in an Italian tertiary care pediatric hospital. The aim was to bridge the possible gap between guidelines recommendations [19] and real-world clinical needs and practices. The script proved efficient and showed the superior efficacy of meropenem (median 95.4%) for this indication, while cefepime and piperacillin–tazobactam had efficacy around 80% (median 81.8% and 80.3%). However, it should be noted that cefepime alone was tested in a small number of strains since it was introduced quite recently in the system. Nevertheless, its clinical utility for Gram-negative BSI is indirectly confirmed by the efficacy in the analysis of combinations where non-tested drugs are considered as resistant. Indeed, the combination of cefepime and piperacillin/tazobactam (and ceftazidime) with amikacin significantly increased the efficacy of the treatment, reaching values like that of meropenem when 95%Cis are considered, suggesting the absence of any clinically relevant difference among the treatment choices [20]. Therefore, the combination used in our center of piperacillin–tazobactam + amikacin (or another aminoglycoside according to local data) could represent a good choice, while the use of cefepime monotherapy might not be as effective as this combination, even if the scarcity of our data could provide distorted information. This combination reaches the same efficacy as meropenem but reduces its use. It is well known indeed that the use of meropenem can be associated with an increasing in resistance selection also towards other antibiotics [21] and is the best drug for “rescue” in the case of clinical failure of initial empirical therapy of febrile neutropenia [22].

The tool we developed provides a crucial, often overlooked, step: the ability to rigorously validate international recommendations against local data before their full implementation. This allows for the easy and frequent creation of updated, locally tailored treatment protocols based on the latest scientific evidence and local epidemiology, resulting in significant time savings and improved efficiency. A key advantage is its capacity for rapid and reproducible data analysis, suggesting its potential to optimize empirical antibiotic therapy across various settings over time.

Our approach can be contextualized within the broader landscape of data-driven empirical therapy models. The Weighted-Incidence Syndromic Combination Antibiogram (WISCA) represents the gold standard for this type of analysis, offering probabilistic predictions based on patient syndromes rather than just organism isolation [13,23,24]. However, the implementation of WISCA requires granular clinical data (often not available in standard laboratory exports) and complex Bayesian hierarchical modeling that is difficult to automate without specialized biostatistical support. In contrast, our tool prioritizes accessibility and reproducibility. By focusing on the validation of guideline-recommended regimens using standard laboratory outputs, we provide a “bridge” solution for centers that cannot yet implement full-scale WISCA models but still require a rigorous method to validate international guidelines against local ecology. While less granular than a syndrome-based model, our script offers the advantage of immediate deployment with zero cost, effectively lowering the barrier to entry for data-driven antimicrobial stewardship. This approach is similar to that of escalating or de-escalating antibiograms derived from multicenter studies [22,25], but applied to a single local reality in a simple and fast way. This ease of use and accessibility, coupled with significant time savings compared to manual evaluation, makes our tool a valuable asset for supporting antimicrobial stewardship programs in resource-limited settings where speed and practicality are essential.

Crucially, the computational validation reported in the results (Table 3) provides empirical evidence supporting the theoretical concerns regarding manual data handling. The Standard Workflow required approximately 150 min of intense manual curation to achieve the same statistical output that the script generated in less than 30 s. Maintaining high-level vigilance for such a prolonged period is physiologically unsustainable for a human operator [17], making the transition to an automated workflow a safety imperative rather than just a time-saving measure. Furthermore, by replacing manual verification with deterministic string-matching algorithms, our tool effectively mitigates the specific risks associated with transcription errors in text-based fields [16], ensuring that local epidemiological insights are not compromised by the latent error rates typical of manual abstraction [14,15].

Our study has some limitations. First, the analysis is retrospective and based on a single-center cohort, which limits the generalizability of our findings. While the script was validated using our data, its performance has not yet been formally tested on external datasets from other hospitals, nor has the direct clinical impact of the tool (such as end-user feedback or its influence on real-time therapeutic decisions) yet been assessed in a prospective setting. Second, we did not conduct a formal study comparing the analysis time with manual evaluation or other existing tools like WISCA. Finally, interpretations were based on current EUCAST rules, but breakpoint definitions for certain organism–drug combinations have evolved over the study period, representing an inherent limitation of retrospective analyses. However, the potential of this approach extends beyond the current cohort; further multicentric studies involving larger populations and different types of infectious syndromes are warranted to confirm the generalizability and utility of this computational workflow in diverse clinical scenarios.

## 4. Materials and Methods

A Python [18] script within the Google Colab environment [26] was developed, using large language models (Google Gemini 3.0 and ChatGPT 5.2 [27,28]) to aid in code generation and debugging. The script was refined, manually edited, and tested to enhance data handling, statistical computations, and result export and visualization. To evaluate the script’s effectiveness, a retrospective observational study was conducted retrieving data from the Microbiology Laboratory database at the IRCCS Istituto Giannina Gaslini (IGG), Genoa, Italy, a tertiary pediatric hospital with extensive experience in the management of cancer and HSCT-related infectious complications. The data covered BSI cases in children receiving antineoplastic chemotherapy or HSCT from 2015 to 2024. Throughout this entire period, the internal protocol for initial empirical antimicrobial therapy for febrile neutropenia remained unchanged, consisting of a combination of piperacillin–tazobactam + amikacin (3 doses in the case of fever of unknown origin). The script was used to evaluate the efficacy of antibiotics against Gram-negative bacteria isolated in BSIs, after exclusion of skin contaminants [29], which is the main goal of empirical therapy in febrile neutropenia. To ensure a comprehensive understanding of our center’s epidemiology, we included all BSI episodes in patients with malignant disease (including autologous and allogeneic HSCT) and not only those observed during neutropenic periods. This approach provides a broader view, considering that individual patients may present with multiple episodes throughout their therapy, including the post-HSCT phases [3]. Data were provided as a spreadsheet file with columns for the microorganism and antibiotic susceptibility results. Antibiotic susceptibility was evaluated by means of Sensititre (Thermo Scientific, Thermo Fisher Diagnostic, Landsmeer, The Netherlands) interpreted according to European Committee on Antimicrobial Susceptibility Testing (EUCAST) rules [30], and categorized as susceptible (S), resistant (R), or not tested (N). The category “Susceptible Dose-Dependent” was considered “S” because our internal protocol adopts the maximum dosage of antibiotics for patients with febrile neutropenia. Interpretations were used instead of the minimal inhibitory concentration (MIC) values due to potential changes in EUCAST’s interpretation of MIC values over the years.

Antibiotics for which susceptibility was collected were chosen based on the following considerations [10,11,22]:

- Piperacillin–tazobactam, ceftazidime, cefepime, and meropenem, recommended as monotherapy for initial empirical therapy of febrile neutropenia.
- Ceftazidime resistance can serve as a marker for extended-spectrum or AmpC beta-lactamases.
- Amikacin, frequently included in combination therapy.
- Ciprofloxacin, a possible alternative to amikacin, especially for patients with impaired renal function, due to its similar anti-Gram-negative spectrum.

Survival within 2 weeks from BSI was also recorded [6].

### 4.1. Analytical Metrics and Statistics

**Figure 1:**
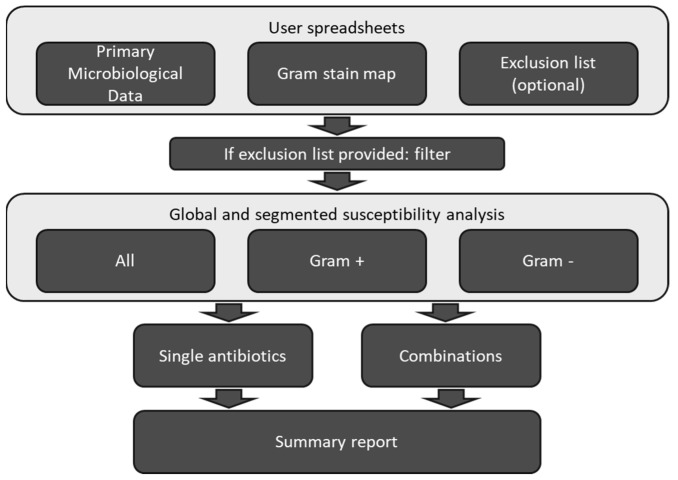
Python script workflow diagram.

Script functionality is summarized in Figure 1. The script operates on a primary spreadsheet detailing microorganism isolates and their corresponding antibiotic susceptibility profiles classified as S, R, or N. To refine the analysis, the user can also provide a separate reference file containing a mapping of microorganism names to their Gram-stain classification and a list of specific isolates to be excluded from the analysis (e.g., common skin contaminants). The core functionality involves a dynamic classification algorithm that automatically assigns a Gram stain (positive or negative) to each microorganism in the primary dataset via partial string matching. For any microorganism not recorded in the reference file, the script interactively prompts the user for its Gram stain, subsequently updating the reference file to ensure persistence and adaptability for future analyses.

A segmented analysis is then performed on Gram-negative isolates. The script calculates two key metrics for both single antibiotics and two-drug combinations. The first metric measures the ratio of S cases to the total number of tested strains (S/S + R). The second metric provides a more conservative estimate, calculated as the ratio of susceptible cases to the total number of records, treating all N cases as R (S/S + R + N). This approach provides a robust lower-bound on efficacy, particularly useful with missing or non-tested data. Both metrics are complemented by a user-defined confidence interval, derived from the Jeffreys interval approximation using beta distribution. To assess differences between monotherapy and combination efficacy, a chi-square test was used.

The script is open-source and can be retrieved at the address https://github.com/Marcello-Mar/Susceptibility-Index-Script (accessed on 2 December 2025). At the same address, an example of Gram-stain map and a common skin contaminants list could be retrieved.

### 4.2. Statistical Validation and Performance Benchmarking

To verify the computational accuracy and operational efficiency of the Python script, a formal validation process was conducted. The entire dataset was analyzed in parallel using a Standard Workflow consisting of manual data curation in a generic spreadsheet followed by statistical analysis using the R software environment version 4.5 (the R Foundation for Statistical Computing). We compared the results of the Python script against this Standard Workflow regarding (i) identification and exclusion of contaminants; (ii) segregation of Gram-negative isolates; (iii) calculation of susceptibility percentages; and (iv) computation of Jeffreys confidence intervals. Additionally, we recorded the total “time-to-result” for both workflows, measured from the raw data extraction to the generation of the final epidemiological report.

## 5. Conclusions

In conclusion, the Python script developed in this study represents a user-friendly, open-source solution for the rapid and standardized analysis of antibiograms, specifically validated in the complex setting of pediatric cancer and HSCT patients. A primary advantage of this tool is its ability to ingest raw data directly from laboratory reports, circumventing the need for manual coding or intermediate review by researchers, which is a process known to introduce systematic errors. By automating this workflow, the tool not only ensures faster analysis but also significantly enhances the reliability of the generated epidemiological data.

Clinically, the application of this script allowed us to quantitatively answer a pivotal question: to what degree can clinicians trust guideline-recommended therapies based on local resistance patterns? Our results validated the high efficacy of meropenem while highlighting the robust potential of piperacillin–tazobactam combined with amikacin as a carbapenem-sparing alternative. Ultimately, by offering a concrete method to validate or challenge international guidelines with real-world data, this tool serves as a scalable asset for antimicrobial stewardship programs, facilitating the transition from static guidelines to dynamic, evidence-based local protocols.

## Figures and Tables

**Table 1:** Isolated Gram-negatives and major antibiotic resistance mechanisms in 237 Gram-negative bloodstream infections during the period 2015–2024.

Microorganisms	Absolute Number	Proportion (% on Total)
Total	237	100
*Enterobacterales*	150	63.3
*Escherichia coli*	74	31.2
*Klebsiella pneumoniae*	32	13.5
*Klebsiella oxytoca*	8	3.4
*Enterobacter cloacae complex*	19	8.0
Other *Enterobacterales* ^1^	17	7.2
*Pseudomonas aeruginosa*	36	15.3
*Pseudomonas oryzihabitans*	6	2.5
*Stenotrophomonas maltophilia*	6	2.5
Other Gram-negatives ^2^	39	16.4
Resistance to antibiotics		
ESBL/AmpC producing Gram-negatives	45	19.0
Carbapenem-resistant Gram-negatives	10	4.2

^1^ Other *Enterobacterales* (n): *Citrobacter* spp. (4), *Enterobacter hormaechei* (4), *Proteus mirabilis* (3), *Pantoea agglomerans* (2), *Campylobacter* spp. (2), *Salmonella* spp. (1), *Serratia* spp. (1). ^2^ Other Gram-negatives (n): *Acinetobacter* spp. (15), Other Pseudomonadales (7), *Stenotrophomonas maltophilia* (6), *Neisseria* spp. (3), Others (8). n = absolute numbers.

**Table 2:** Efficacy of antibiotics against 196 Gram-negative strains measured by the script. 95% CI = 95% confidence interval.

Monotherapy				Combination (At Least 1 Drug Susceptible)			
Antibiotic	Tested Strains *, n	Efficacy		Antibiotic Combination	Tested Strains for at Least One Drug, n	Efficacy	
		Median	95% CI			Median	95% CI
Meropenem	216	95.4	91.7–97.8	Meropenem + amikacin	217	96.3	92.9–98.4
Cefepime	33	81.8	64.5–93	Piperacillin/tazobactam + amikacin	217	95.4	91.7–97.8
Piperacillin/tazobactam	203	80.3	74.1–85.5	Ceftazidime + amikacin	215	94.5	90.6–97.1
Ceftazidime	210	76.2	69.8–81.8	Cefepime + amikacin	205	93.5	89.4–96.4
Amikacin	216	93.5	89.4–96.4	Piperacillin/tazobactam + ciprofloxacin	214	87.6	82.4–91.6
Ciprofloxacin	215	69.3	62.7–75.4	Ceftazidime + ciprofloxacin	215	83.9	78.4–88.6
				Cefepime + ciprofloxacin	163	73.1	66.7–78.9

* Total tested strains vary for each antibiotic as not all isolates were tested against all agents in the retrospective dataset.

**Table 3:** Validation of the Python script versus Standard Workflow (Spreadsheet + R).

Feature/Metric	Standard Workflow (Spreadsheet + R)	Python Script (Automated)	Concordance/Outcome
Susceptibility Calculation	R (Manual coding required)	Automated	100% Match
95% Confidence Interval	R (Manual coding required)	Automated (Jeffreys)	100% Match
Gram-Stain Classification	Manual sorting in spreadsheet	Automated (Dictionary-based)	N/A
Exclusion of Contaminants	Manual filtering in spreadsheet	Automated (Exclusion list)	N/A
Total Processing Time	~150 min	~ 30 s	>99% time saving

## Data Availability

The Python script developed for this study is open-source and publicly available in the GitHub repository (latest version Release Candidate 6). The clinical data presented in this study are available upon request from the corresponding author.
